# Chitosan Hydrogel Structure Modulated by Metal Ions

**DOI:** 10.1038/srep36005

**Published:** 2016-10-25

**Authors:** Jingyi Nie, Zhengke Wang, Qiaoling Hu

**Affiliations:** 1 MoE Key Laboratory of Macromolecular Synthesis and Functionalization, Department of Polymer Science and Engineering, Zhejiang University, Hangzhou, China; 2 Key Laboratory of Adsorption and Separation Materials & Technologies of Zhejiang Province, Hangzhou, China

## Abstract

As one of the most important polysaccharide, chitosan (CS) has generated a great deal of interest for its desirable properties and wide applications. In the utilization of CS materials, hydrogel is a major and vital branch. CS has the ability to coordinate with many metal ions by a chelation mechanism. While most researchers focused on the applications of complexes between CS and metal ions, the complexes can also influence gelation process and structure of CS hydrogel. In the present work, such influence was studied with different metal ions, revealing two different kinds of mechanisms. Strong affinity between CS and metal ions leads to structural transition from orientation to multi-layers, while weak affinity leads to composite gel with *in-situ* formed inorganic particles. The study gave a better understanding of the gelation mechanism and provided strategies for the modulation of hydrogel morphology, which benefited the design of new CS-based materials with hierarchical structure and facilitated the utilization of polysaccharide resources.

Polysaccharide has gained tremendous attention as renewable resource, providing opportunity in sustainable development^1^. Chitosan (CS), a polysaccharide obtained by the deacetylation of chitin, has received considerable attention. With applications pervading advanced industry and research^2^,^3^,^4^,^5^,^6^, CS has been utilized in the preparation of various materials^7^. CS is becoming an increasingly important natural polymer because of its unique properties like biodegradability, biocompatibility and bioactivity^8^,^9^,^10^, in addition to physical and mechanical properties^9^,^11^. Therefore, the utilization of CS has both environmental and practical importance.

In the utilization of CS material, hydrogels is a major and vital branch, since CS hydrogel not only represents a very important form of CS materials, but also provides a vital approach for the preparation of dry CS-based materials such as scaffold^12^,^13^, wound dressing^14^,^15^, and bone fracture fixation devices^16^,^17^,^18^. The control of hydrogel structure, has been extensively studied to meet various needs in specialized fields. Although the study of structure and interactions in CS hydrogels sheds light on formation mechanisms on molecular level^19^,^20^, the construction of highly sophisticated microstructure remains challenging^21^. Thus the understanding of gelation mechanisms possesses fundamental importance for the design of hydrogel with enhanced mechanical and functional performance. The basic way to prepare a CS hydrogel is solubilisation of CS in an acidic aqueous medium. The system lays the foundations of CS material preparation in many aspects, including fabrication of sophisticated organization^21^,^22^,^23^,^24^ and preparation of CS-based composite materials^25^,^26^,^27^.

It is well known that CS demonstrates the affinity towards many metal cations. There are abundant amino groups and hydroxyl groups in the macromolecule. Metal cations can be adsorbed by chelation on amino groups of CS due to the free electron doublet on nitrogen, and hydroxyl groups (especially in the C-3 position) may also contribute to sorption^28^. While most researchers focused directly on the applications of complexes between CS and metal ions, like decontamination of effluents^29^, catalysis industry^30^, advanced composite soft materials^31^,^32^, and noble metals reclamation^33^, few attention was paid on the design of novel hydrogel materials with hierarchical structure taking advantage of the complexes. In addition to the interaction of metal ions and CS on molecular level, it has been reported that the introduction of metal ions can influence the structure of *in-situ* prepared CS hydrogel^34^,^35^. The influence, compared with pure CS material, was revealed by more sophisticated morphology of the resultant composite CS material. However, characteristics of such influence and the mechanism behind still remained in the dark.

In the present work, the influence of metal ions on CS hydrogel structure was studied. Since the affinity between CS and different metal ions are not identical, different kinds of metal ions were selected in the study, and the corresponding mechanisms were discussed. Based on these mechanisms, design and modulation strategies of CS-based hydrogel were proposed, which will hopefully facilitated the development of CS materials and the utilization of polysaccharide resource.

## Results and Discussion

### Structural characters of CS hydrogel

CS becomes a polyelectrolyte because of the protonation of –NH_2_ groups^36^, and the CS solution can be transformed into hydrogel when it comes in contact with alkali. CS hydrogel coagulated by bath with low *c*(OH^−^) had randomly located voids and very low modulus, which would not be discussed further in this work. When the gelation process is powered by high *c*(OH^−^), the gelation process possesses a layer-wise character due to the equipotential surface of *c*(OH^−^)^21^,^23^. This character brings spatiotemporal sequence to the system (Supplementary Fig. S1), which endows orientation along the direction of OH^−^ diffusion^23^.

**Figure 1:**
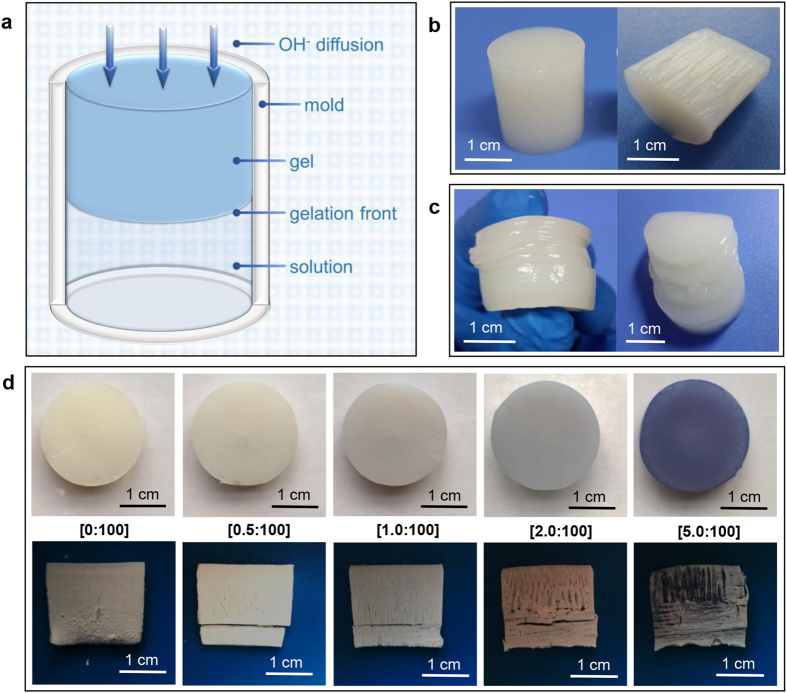
Influence of Cu^2+^ on CS hydrogel structure. (**a**) Schematic illustration of the formation of CS hydrogel; (**b**) Typical structure of CS hydrogel: oriented fibrous structure; (**c**) Multi-layered structure in CS hydrogel; (**d**) The cross section (top panel, native wet state) and longitudinal section (bottom panel, freeze-dried) of copper-CS hydrogels with different molar ratios of Cu^2+^ and amino group.

A one-dimensional model was used to demonstrate the structural characters of CS hydrogel (Fig. 1a). The oriented fibrous structure of CS hydrogel was shown in Fig. 1b and schematically illustrated in Supplementary Fig. S2a. The mechanism had been reported and could be summarized as follows. Phase separation happened in the primary hydrogel layer. Then during the layer-wised gelation process, macromolecules at the gelation front were inclined to rearrange below CS-rich zones of the previous layer due to entanglement in the gel-sol interface^23^.

CS hydrogel can also form multi-layered structure (Fig. 1c and Supplementary Fig. S2b). The formation of layers can be explained by the Liesegang Ring Phenomenon, which is caused by periodical precipitation in the supporting medium by the encounter of an inner and an outer electrolyte^37^,^38^. Supersaturation theory is one of the classic Liesegang Ring Phenomenon theories^39^. Based on the theory, a precipitate of inner and outer electrolyte is not formed immediately, and the product remains in supersaturated solution. When it precipitates a short distance behind the diffusion front, the product in solution diffuses toward the nuclei, and the inner electrolyte was also absorbed by the precipitate. Thus a “clear space” was formed. When precipitate further formed after the clear space, the boundary of two bands came into being as a result. For the system of CS solution, the diffusion of supersaturated product towards precipitates was essentially the chain condensation (Supplementary Fig. S3). However, pure CS system can only form one or two layers spontaneously, which is much less than typical Liesegang Ring Phenomenon. This is because the diffusion of supersaturated product was much more difficult than typical inorganic product, since the product corresponded to macromolecules in this case. The diffusion difficulty originated from the entanglement and limited mobility of macromolecules. As introduced above, the core principle of layer formation is the chain condensation. Thus, in order to fabricate layers in CS hydrogel, signals were often given by alternate process to induce chain condensation^21^,^24^. Specifically speaking, the gelation was manipulated by “on-off” program. Sol-gel transition happened in the “on-stage” and paused in the “off-stage”. During the pause, the entangled chains at gel-sol interface disentangled, leading to chain condensation. When sol-gel transition proceeded in the next “on-stage”, two spatially separated layers came into being.

### Hydrogel structure transition from orientation to multi-layers by metal ions with strong affinity

Copper was focused in many studies of the chelation mechanisms of CS and metal ions^28^. Although several contradictory hypotheses have been proposed for the interpretation of copper uptake, it is well known that CS has strong affinity to Cu^2+^ ions. So the introduction of Cu^2+^ might influence the behaviour of CS, and further influence the structure of CS hydrogel. In order to study the influence, an *in-situ* precipitation method was employed in this work. A metal ions-CS solution was formed first, in which CS was solubilized due to protonation of partial amino groups, while Cu^2+^ form complex with the free –NH_2_ and –OH. Second, Cu^2+^-CS hydrogel was *in-situ* formed with the diffusion of OH^−^.

When other parameters were fixed, the solution samples were clear with *n*(Cu^2+^)/*n*(-NH_2_) lower than 5:100 (molar ratios of Cu^2+^ and amino groups), and became cloudy when *c*(Cu^2+^) kept increasing (Supplementary Fig. S4). This was because the crosslinking effect of Cu^2+^ decreased the solubility of CS. The formation of complex in solution was validated by the increase of viscosity with rise of *c*(Cu^2+^) (Supplementary Fig. S5), and the decrease of viscosity with rise of *c*(H^+^) (Supplementary Fig. S6). When comparing copper-CS hydrogel with different *n*(Cu^2+^)/*n*(-NH_2_), the result indicated that the increase of *c*(Cu^2+^) enhanced the mechanical strength of CS hydrogel (Supplementary Fig. S7a). This could also be understood in view of the ionic cross-linking.

**Figure 2:**
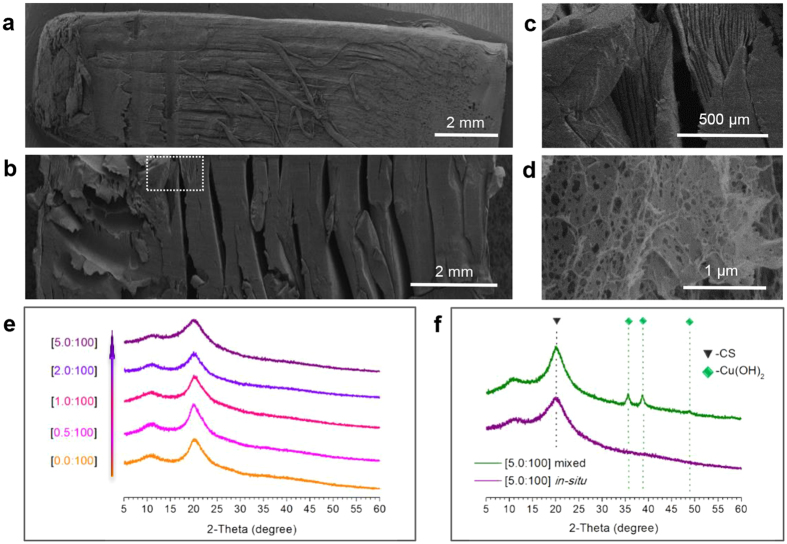
Microscopic structure of copper-CS hydrogel. (**a**) SEM image of freeze-dried CS hydrogel sample; (**b–d**) SEM images of freeze-dried copper-CS hydrogel samples, (**c**) corresponds to the marked area in (**b**), *n*(Cu^2+^)/*n*(-NH_2_) = 5.0:100; (**e**) XRD profiles of copper-CS samples with different molar ratios of Cu^2+^ and amino group; (**f**) XRD profiles of copper-CS sample prepared by *in-situ* precipitation and Cu(OH)_2_-CS sample prepared by mixing, *n*(Cu)/*n*(-NH_2_) = 5.0:100.

Besides the change of strength, it was very interesting that the structure of hydrogel also changed with the rise of *c*(Cu^2+^) (Fig. 1d), and the change possessed specific patterns (schematically illustrated in Supplementary Fig. S8). (1) When *n*(Cu^2+^)/*n*(-NH_2_) = 0, hydrogel showed typical oriented fibrous structure (Fig. 2a). With the rise of *c*(Cu^2+^), the presence of fibrous structure was retained, but the space between fibres increased apparently (Supplementary Fig. S9). (2) Formation of layers appeared with the rise of *c*(Cu^2+^). Moreover, with higher *n*(Cu^2+^)/*n*(-NH_2_) value, the formation of layer happened earlier and closer to the coagulation-system interface. (3) Multi-layered structure appeared with high *n*(Cu^2+^)/*n*(-NH_2_) value, and the number of layers dramatically increased compared with pure CS hydrogel (Fig. 2b,c). The change of hydrogel structure can be summarized as the spontaneous transition from oriented fibres to multi-layers.

Reasons behind such transition were investigated and discussed. The spontaneous formation of layers showed resemblance with the Liesegang Ring Phenomenon. In the case of pure CS hydrogel, as mentioned above, multi-layered structures cannot be formed spontaneously because of the limited mobility of macromolecules. However, with the introduction of metal ions, there is another kind of inner electrolyte in the system, *i.e.* the Cu^2+^. Based on the supersaturation theory mentioned above, Cu^2+^ could form precipitation of Cu(OH)_2_ when encountered the outer electrolyte (OH^−^). Compared with macromolecues, the supersaturated Cu(OH)_2_ product diffused much easier, and might form multi-precipitate bands under desired condition. So one possible hypotheses is that the formation of Cu(OH)_2_ precipitate band acted as nuclei for the polyelectrolyte, and promoted the separation of layers by creating “clear space” of CS.

However, when the microscopic morphology of copper-CS hydrogel was investigated, no inorganic crystalline nor precipitate band was observed in the sample, (Fig. 2d, Supplementary Fig. S10). The hypotheses was further investigated by XRD. The profiles of all samples showed characteristic diffraction peak of CS (2*θ* = 20°), but none of them showed diffraction peaks of Cu(OH)_2_ or any other inorganic components (Supplementary Fig. S11). As a comparison group, mixture of CS and Cu(OH)_2_ was prepared with *n*(Cu(OH)_2_)/*n*(-NH_2_) = [5:100], which was the same as the copper-CS hydrogel with the highest *n*(Cu^2+^)/*n*(-NH_2_) value. Results showed that, XRD profile of the mixture clearly showed diffraction peaks of Cu(OH)_2_. The results indicated that the absence of Cu(OH)_2_ was not due to the detection limit of XRD or low amount of inorganic component. Cu^2+^ ions did not exist as Cu(OH)_2_ in the gel, nor did they induced layer formation of CS as multi-precipitate bands. On the other hand, it had been reported that the complex between Cu^2+^ and CS were reserved after co-precipitation^40^. These results indicated that, Cu^2+^ did not fom precipitates as Liesegan Rings under conditions of this study. So the hypotheses mentioned above could be excluded.

**Figure 3:**
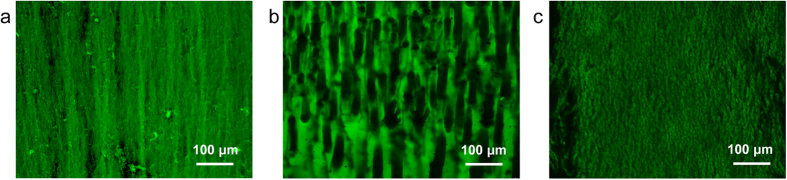
Morphology of CS-based hydrogel samples in native wet state. Confocal laser scanning microscopy image of (**a**) CS hydrogel sample, (**b**) copper-CS hydrogel sample (*n*(Cu^2+^)/*n*(-NH_2_) = 5.0:100), and (**c**) calcium-CS hydrogel sample (*n*(Ca^2+^)/*n*(-NH_2_) = 40.0:100).

Thus it is highly possible and reasonable that Cu^2+^ ions derectly influenced the behavior of CS macromolecules. The influece was then investigated from the relationship between strucutral chage and *c*(Cu^2+^). It has been observed that, size of voids in hydrogel increased with the rise of *c*(Cu^2+^). To further exclude the interference of drying process, CS hydrogel and copper-CS hydrogel were observed in native wet state (Fig. 3a,b). Comparing the oriented part of pure CS and copper-CS samples, the latter showed more compact polymer zones with larger voids (Fig. 3b). Due to the adhesion between the system and the side wall of single opening mold, the system would not show lateral macroscopic shrinking. So the polymer zone compactness and voids size reflected the shrinking tendency in the hydrogel. The results above indicated that copper-CS system had greater tendency of volume shrinking during gelation. This could be accounted by the increased inter/intramolecular interaction among CS chains after bound by Cu^2+^.

**Figure 4:**
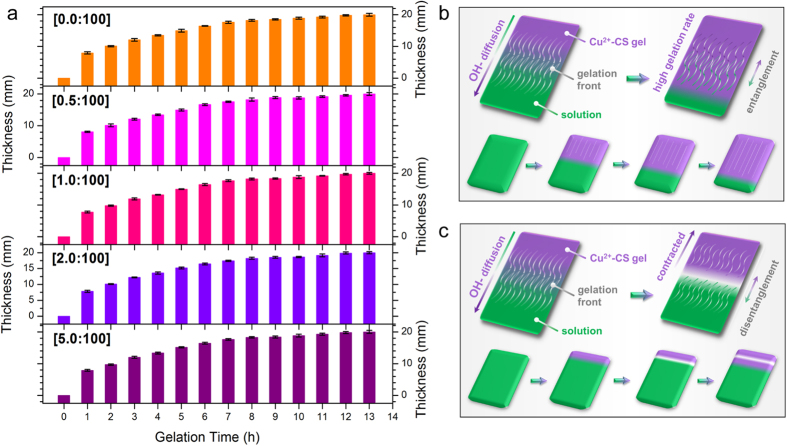
Gelation rate and gelation process of Cu^2+^-CS solution. (**a**) Relationship between gel thickness and gelation time with different molar ratios of Cu^2+^ and amino group, error bars indicate standard errors for n = 3; (**b,c**) Schematic illustration of gelation process of Cu^2+^-CS solution: (**b**) formation of oriented fibrous structure, (**c**) formation of multi-layered structure.

Besides intrinsic difference between CS and Cu^2+^-CS systems, another factor has to be considered when investigating structrual transition, *i.e.* the gelation rate. The gelation rate has decisive influence on CS hydrogel structure^23^. As mentioned above and demonstrated in Supplementary Fig. S12, the gelation process has spatiotemporal sequence. Moreover, the gelation rate varied at different distance, due to the increasing diffusion distance to the OH^−^ source. The most intriguing thing is that, the structural transition from orientation to multi-layers also happened at different distance to the coagulation-system interface. This indicated that the structural transition could be essentially related to the decrease of gelation rate. As shown in Fig. 4a, for systems with different *n*(Cu^2+^)/*n*(-NH_2_), the relationships between gel thickness and gelation time were almost identical. This was because the difference of *c*(Cu^2+^) was negligible due to high consentration of OH^−^. It is noteworthy that, the gelation rates of all copper-CS systems were in good accordance with the pattern mentioned above, which decreased with the rise of diffusion distance.

When taking the two factors above into consideration, the mechanism of layer formation and structural transition can be summarized as follows. Macromolecues entangelment exixted on the gel-sol interface. Cu^2+^ and CS formed strong complex, leading to increased tendency of volume shrinking of polymer zones compared with pure CS system. When gelation front was near the system-coagulation interface (Fig. 4b), the gelation rate was high, and the disentanglement was not sufficient in gel-sol interface. Thus upper gelation unit right behind gelation front still possessed influence on the next unit^23^. So in this stage, orientation was preserved and the increased shrinking tendency led to larger voids between fibres. When gelation front was distant from the system-coagulation interface (Fig. 4c), the gelation rate decreased apparently. It took longer for CS chains at the gelation front to lose mobility thus disentanglement could happen. The shrinking tendency created a contraction at the gel-sol interface and enhanced the disentanglement of macromolecules. Thus, a “clear space” was formed. With further diffusion of OH^−^, gelation proceeded, creating two layers. The occurrence of disentanglelemt corrsponded to the “off-stage” discussed above. Only in this case, the switch between “on-stage” and “off-stage” was spontaneous, controlled by the competetion between gelation rate and disentanglement.

### Unchanged hydrogel structure with weak affinity metal ions

As discussed above, CS has strong affinity with Cu^2+^ ions. Since the complex between metal ions and macromolecules is a crucial factor for structural transition, what the influence will be if CS has weak affinity with the metal ions in the system? It has been observed that CS has very limited affinity with alkaline-earth metals due to the absence of d and f unsaturated orbitals (unlike transition metals)^41^. So calcium ions (Ca^2+^) was chosen to investigate this influence. Ca^2+^ could also be fabricated into calcium-CS hydrogel via *in-situ* precipitation, and the maximum *n*(Ca^2+^)/*n*(-NH_2_) value is much higher than that of Cu^2+^ ions (Supplementary Fig. S13a). Because the weak complex did not lead to evident ionic cross-linking compared with Cu^2+^. This was validated by the mechanical strength of calcium-CS hydrogel. The introduction of Ca^2+^ did not exert much influence on the strength of CS hydrogel (Supplementary Fig. S7b).

**Figure 5:**
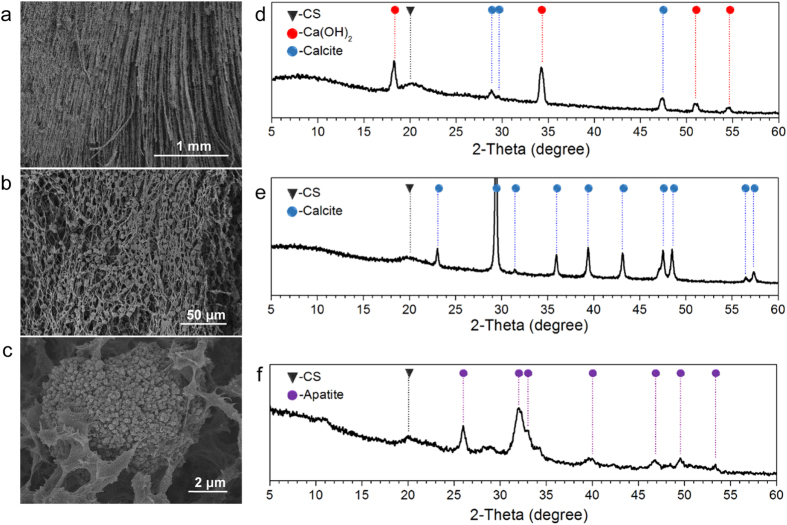
Microscopic structure of calcium-CS hydrogel. (**a–c**) SEM images of freeze-dried calcium-CS hydrogel sample; (**d**) XRD profile of calcium-CS hydrogel sample prepared by *in-situ* precipitation; (**e,f**) XRD profiles of calcium salt-CS hydrogel samples transferred after *in-situ* precipitation: (**e**) calcite-CS hydrogel sample, and (**f**) apatite-CS hydrogel sample. *n*(Ca^2+^)/*n*(-NH_2_) = 40.0:100.

Observation of morphology showed that calcium-CS hydrogel had oriented fibrous structure (Fig. 5a), and did not show transition to layered structure (Supplementary Fig. S13b). Small particles were embedded in the fibrous matrix (Fig. 5b,c and Supplementary Fig. S14a,b). XRD tests proved that these particles were inorganic component originated from Ca^2+^. XRD profile showed characteristic diffraction peaks of CS, Ca(OH)_2_ and calcite (Fig. 5d). Ca(OH)_2_ was formed due to the encounter of OH^−^, and partly transferred to calcite after contacted with CO_2_ in air during the subsequent operations.

More importantly, morphology of hydrogel in native wet state showed that, calcium-CS hydrogel did not present increased tendency of volume shrinking (Fig. 3c, Supplementary Fig. 14c). The oriented fibrous structure was similar to pure CS hydrogel. Only in this case, the fibres appeared to be “curly”, which was caused by the embedded inorganic particles. The formation mechanism of the typical structure in calcium-CS hydrogel was summarized as follows. Ca^2+^ has weak affinity with CS, and turned to precipitate when encountered OH^−^. Thus the introduction of Ca^2+^ did not directly influenced the gelation behavior of CS.

### Influence of different metal ions and structrue design

**Figure 6:**
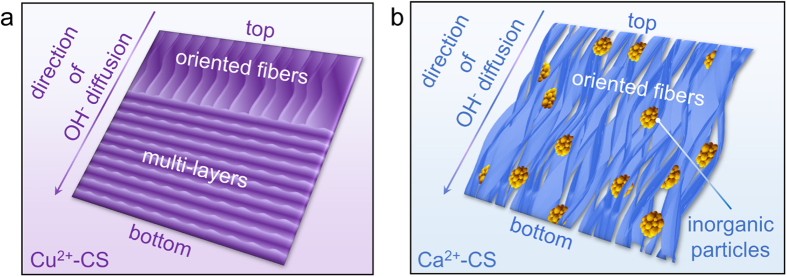
Schematic illustration of the typical morphology of copper-CS hydrogel and calcium-CS hydrogel. (**a**) Copper-CS hydrogel; (**b**) Calcium-CS hydrogel.

Typical structures and formation of copper-CS hydrogel and calcium-CS hydrogel were schematically illustrated in Fig. 6 and Supplementary Fig. S15, respevctively. Pure CS hydrogel, copper-CS hydrogel and calcium-CS hydrogel were all real gel materials (Supplementary Fig. S16). The introduction of metal ions that has strong affinity with CS, such as Cu^2+^ ions, leads to structural transition directly. This could be the design basis of metal-CS complex hydrogel with sophisticated structure and enhanced strength. These materials have potential values in applications such as copper-based fungicides^42^, redox catalysts on the basis of CS matrix^30^, pre-concentration of inorganic arsenic anions^43^, and urea uptake^42^. On the other hand, the influence was quite different for metal ions that has weak affinity with CS, which was also very beneficial. By *in-situ* precipitation, inorganic particles could be incorporated in hydrogel matrix with oriented fibrous structure. Such morphology cannot be achieved by simple blending nor layer-by-layer process^44^. Moreover, these inorganic particles can be completely transferred to calcite with the support of CO_3_^2−^ (Fig. 5e), or transferred to apatite with the support of H_2_PO_4_^−^ (Fig. 5f). This provided a novel approach for the fabrication of various calcium salts-CS composite materials, which has great potential in the field of bone repair.

## Conclusion

In summary, the introduction of different metal ions exerted different influence on the CS hydrogel structure. The introduction of metal ions that has strong affinity with CS, such as Cu^2+^ ions, increases the volume shrinking tendency of polymer zons due to ionic crosslinking effect. The shrinking tendency creates contraction at the gel-sol interface, and could lead to structural transition to multi-layers with proper gelation rate. While metal ions that has weak affinity with CS, such as Ca^2+^ ions, tends to form precipitates themselves when encountered OH^−^. This leads to the incorporation of inorganic particles, without chainging the structrual characteristics of CS hydrogel. The study on the influence of metal ions on CS hydrogel structure gave a better understanding of the gelation mechanism, and provided strategies for the modulation of hydrogel morphology. This benefited the design of new CS-based materials and facilitated the utilization of polysaccharide resources.

## Methods

### Materials and reagents

CS was purchased from Zhejiang Gold Shell Pharmaceutical Co. Ltd. The average viscosity molecular weight (M*η*) of CS was 5.63 × 10^5^ Da, and degree of deacetylation (DD) was 91%. Fluorescein iso-thiocyanate (FITC) was purchased from Sigma Chemical Company. FITC labelled CS (FITC-CS) was prepared following the process reported in literature^45^. Sodium hydroxide (NaOH), acetic acid, copper chloride (CuCl_2_), calcium chloride (CaCl_2_), and copper (II) hydroxide (Cu(OH)_2_) were purchased from Sinopharm Chemical Reagent Co., Ltd. and were of analytical reagent grade.

### Preparation of CS hydrogel samples

CS hydrogel sample with oriented fibrous structure was prepared as follow. CS solution with the final CS concentration as 5 *wt.*%, was prepared by dissolving CS powder in 2 *vol.*% acetic acid aqueous solution. Coagulation bath was 10 *wt.*% NaOH aqueous solution. The volume of coagulation bath was much larger than that of CS solution, so the *c*(OH^−^) could be considered as constant. The volume of CS solution for one sample varied slightly with different kinds of molds used in this study, and was in the range of 5–10 mL. For one CS sample, the volume of coagulation bath was 1000 mL. CS solution was filled in a single opening mold and immersed in the coagulation bath till the gelation was completed. CS hydrogel with multi-layered structure was prepared as follows. The single opening mold with CS solution was first immersed in coagulation bath. Then after a certain interval, the gelation process was interrupted by transferring the mold into a deionized water bath for a same interval. The sequence was repeated 5 times. After gelation, resultant gel samples were rinsed repeatedly to be neutral with deionized water, and monitored by a pH meter (PHS-3C, INESA Scientific Instrument Co., Ltd).

### Preparation of copper-CS and calcium-CS hydrogel samples

Copper-CS complex hydrogels were prepared via an *in-situ* precipitation method. First, a Cu^2+^-CS solution was prepared, with the final CS concentration as 5 *wt.*%. CuCl_2_ powder was used as the source of Cu^2+^ in the system, which was added to pure CS solution to form the Cu^2+^-CS solution. Samples with different concentration of Cu^2+^ were prepared, and the concentration was denoted as the molar ratios between Cu^2+^ and amino group. A single opening mold was used to fabricate the hydrogel. Then the solution was filled in the single opening mold and immersed in the coagulation bath till the gelation was completed. After that, resultant complex CS gel was unloaded from the mold, and were washed with deionized water repeatedly to be neutral. Calcium-CS complex hydrogels were prepared by similar procesure.

### Morphology observation

Hydrogel samples were studied by scanning electron microscopy (SEM, HITACHI S-4800), and confocal laser scanning fluorescence microscope (CLSM, Leica TCS SP5). For SEM observation, CS hydrogels were freeze-dried and then gold-sprayed for conductance. For confocal fluorescence microscopy characterization, hydrogels were prepared with FITC-CS and kept wet during observation.

### X-ray measurements

Wide-angle X-ray diffraction profiles were collected at room temperature, with a Bruker AXS D8 Advance X-ray diffractometer (40 kV, 34 mA, Cu Kα radiation, k = 1.5406 Å) scanned at 2 degree (2*θ*) s^−1^ in the 2*θ* range 5–60°. Hydrogel smaples were freeze-dried and then prepared to be powder before characterization.

### Hydrogel formation rate

Hydrogel formation rate was calculated by reaction time and gel thickness^23^. The reaction started as soon as the mold was immersed in the coagulation bath. The gel thickness was the distance between the system-coagulation interface and the gel-sol interface, and was determined by a vernier caliper. The experiments were repeated three times.

## Additional Information

**How to cite this article**: Nie, J. *et al*. Chitosan Hydrogel Structure Modulated by Metal Ions. *Sci. Rep.* **6**, 36005; doi: 10.1038/srep36005 (2016).

## Supplementary Material

Supplementary Information

## References

[b1] Gandini A. Polymers from renewable resources: a challenge for the future of macromolecular materials. Macromolecules 41, 9491–9504 (2008).

[b2] Aider M. Chitosan application for active bio-based films production and potential in the food industry: Review. Lwt-Food Sci. and Technol. 43, 837–842 (2010).

[b3] Fang J., Zhang K., Jia J., Wang Z. & Hu Q. Preparation and characterization of N-phthaloyl-chitosan-g-(PEO-PLA-PEO) as a potential drug carrier. Rsc Adv. 5, 99418–99424 (2015).

[b4] Jia J. . Monitoring layer-by-layer self-assembly process of natural polyelectrolytes by fluorescent bioconjugate with aggregation-induced emission characteristic. J. Mater. Chem. B 2, 8406–8411 (2014). 10.1039/c4tb01158a 32262011

[b5] Zhang K. . Preparation of chitosan/hydroxyapatite guided membrain used for periodontal tissue regeneration. Chin. J. Polym. Sci. 28, 555–561 (2010).

[b6] Zhang K. . One-pot synthesis of chitosan-g-(PEO-PLLA-PEO) via “click” chemistry and “SET-NRC” reaction. Carbohydr. Polym. 90, 1515–1521 (2012). 22944410 10.1016/j.carbpol.2012.07.023

[b7] Ravi Kumar M. N. V. A review of chitin and chitosan applications. React. Funct. Polym. 46, 1–27 (2000).

[b8] Rinaudo M. Chitin and chitosan: Properties and applications. Prog. Polym. Sci. 31, 603–632 (2006).

[b9] Huang X. . Using absorbable chitosan hemostatic sponges as a promising surgical dressing. Int. J. Biol. Macromol. 75, 322–329 (2015). 25661881 10.1016/j.ijbiomac.2015.01.049

[b10] Huang X., Jia J., Wang Z. & Hu Q. A novel chitosan-based sponge coated with self-assembled thrombin/tannic acid multilayer films as a hemostatic dressing. Chin. J. Polym. Sci. 33, 284–290 (2015).

[b11] Sun Y. F., Li Y. L., Nie J. Y., Wang Z. K. & Hu Q. L. High-strength Chitosan Hydrogels Prepared from LiOH/Urea Solvent System. Chem. Lett. 42, 838–840 (2013).

[b12] Madihally S. V. & Matthew H. W. T. Porous chitosan scaffolds for tissue engineering. Biomaterials 20, 1133–1142 (1999). 10382829 10.1016/s0142-9612(99)00011-3

[b13] Zhang J. . Preparation and characterization of bionic bone structure chitosan/hydroxyapatite scaffold for bone tissue engineering. J. Biomater. Sci. Polym. Ed. 25, 61–74 (2014). 24053536 10.1080/09205063.2013.836950

[b14] Boateng J. S., Matthews K. H., Stevens H. N. E. & Eccleston G. M. Wound healing dressings and drug delivery systems: A review. J. Pharm. Sci. 97, 2892–2923 (2008). 17963217 10.1002/jps.21210

[b15] Jayakumar R., Prabaharan M., Kumar P. T. S., Nair S. V. & Tamura H. Biomaterials based on chitin and chitosan in wound dressing applications. Biotechnol. Adv. 29, 322–337 (2011). 21262336 10.1016/j.biotechadv.2011.01.005

[b16] Pu X. M. . Fabrication of chitosan/hydroxylapatite composite rods with a layer-by-layer structure for fracture fixation. J. Biomed. Mater. Res. B 100B, 1179–1189 (2012). 10.1002/jbm.b.31961 22454303

[b17] Wang Z., Hu Q. & Wang Y. Preparation of chitosan rods with excellent mechanical properties: one candidate for bone fracture internal fixation. Sci. Chi. Chem. 54, 380–384 (2011).

[b18] Wang Z. & Hu Q. Preparation and properties of three-dimensional hydroxyapatite/chitosan nanocomposite rods. Biomed. Mater. 5, 1748–6041 (2010). 10.1088/1748-6041/5/4/045007 20603528

[b19] Berger J., Reist M., Mayer J. M., Felt O. & Gurny R. Structure and interactions in chitosan hydrogels formed by complexation or aggregation for biomedical applications. Eur. J. Pharm. Biopharm. 57, 35–52 (2004). 14729079 10.1016/s0939-6411(03)00160-7

[b20] Berger J. . Structure and interactions in covalently and ionically crosslinked chitosan hydrogels for biomedical applications. Eur. J. Pharm. Biopharm. 57, 19–34 (2004). 14729078 10.1016/s0939-6411(03)00161-9

[b21] Ladet S., David L. & Domard A. Multi-membrane hydrogels. Nature 452, 76–U76 (2008). 18322531 10.1038/nature06619

[b22] Nie J., Wang Z., Zhang K. & Hu Q. Biomimetic multi-layered hollow chitosan-tripolyphosphate rod with excellent mechanical performance. Rsc Adv. 5, 37346–37352 (2015).

[b23] Nie J. . Orientation in multi-layer chitosan hydrogel: morphology, mechanism, and design principle. Sci. Rep. 5, 7635 (2015). 25559867 10.1038/srep07635 PMC4284508

[b24] Yan K. . Coding for hydrogel organization through signal guided self-assembly. Soft Matter 10, 465 (2014). 24652449 10.1039/c3sm52405a

[b25] Yao H. B., Fang H. Y., Tan Z. H., Wu L. H. & Yu S. H. Biologically Inspired, Strong, Transparent, and Functional Layered Organic-Inorganic Hybrid Films. Angew. Chem. Int. Ed. 49, 2140–2145 (2010). 10.1002/anie.200906920 20187052

[b26] Yao H. B., Tan Z. H., Fang H. Y. & Yu S. H. Artificial Nacre-like Bionanocomposite Films from the Self-Assembly of Chitosan-Montmorillonite Hybrid Building Blocks. Angew. Chem. Int. Ed. 49, 10127–10131 (2010). 10.1002/anie.201004748 21110359

[b27] Sahiner N. Soft and flexible hydrogel templates of different sizes and various functionalities for metal nanoparticle preparation and their use in catalysis. Prog. Polym. Sci. 38, 1329–1356 (2013).

[b28] Guibal E. Interactions of metal ions with chitosan-based sorbents: a review. Separ. Purif. Technol. 38, 43–74 (2004).

[b29] Barakat M. A. & Schmidt E. Polymer-enhanced ultrafiltration process for heavy metals removal from industrial wastewater. Desalination 256, 90–93 (2010).

[b30] Guibal E. Heterogeneous catalysis on chitosan-based materials: a review. Prog. Polym. Sci. 30, 71–109 (2005).

[b31] Sun Z. . Multistimuli-Responsive, Moldable Supramolecular Hydrogels Cross-Linked by Ultrafast Complexation of Metal Ions and Biopolymers. Angew. Chem. Int. Ed. 54, 7944–7948 (2015). 10.1002/anie.201502228 26012538

[b32] Zhang Z. . The *in-situ* synthesis of Ag/amino acid biopolymer hydrogels as mouldable wound dressings. Chem. Commun. 51, 15862–15865 (2015). 10.1039/c5cc05195a 26377374

[b33] Inoue K., Yamaguchi T., Iwasaki M., Ohto K. & Yoshizuka K. Adsorption of Some Platinum Group Metals on Some Complexane Types of Chemically Modified Chitosan. Separ. Sci. Technol. 30, 2477–2489 (1995).

[b34] Ke J., Wang Z., Li Y., Hu Q. & Feng J. Ferroferric oxide/chitosan scaffolds with three-dimensional oriented structure. Chin. J. Polym. Sci. 30, 436–442 (2012).

[b35] Qu J. . The preparation and characterization of chitosan rods modified with Fe^3+^ by a chelation mechanism. Carbohydr. Res. 346, 822–827 (2011). 21382612 10.1016/j.carres.2011.02.006

[b36] Rinaudo M., Pavlov G. & Desbrieres J. Influence of acetic acid concentration on the solubilization of chitosan. Polymer 40, 7029–7032 (1999).

[b37] Droz M. Recent theoretical developments on the formation of Liesegang patterns. J. Stat. Phys. 101, 509–519 (2000).

[b38] Hu Q. L. Research on chitosan medical material with bionic multi-layered structure. Ph.D thesis, Zhejiang University (2004).

[b39] Stern K. H. The Liesegang phenomenon. Chem. Rev. 54, 79–99 (1954).

[b40] Kucherov A. V., Kramareva N. V., Finashina E. D., Koklin A. E. & Kustov L. M. Heterogenized redox catalysts on the basis of the chitosan matrix: 1. Copper complexes. J. Mol. Catal. A-Chem. 198, 377–389 (2003).

[b41] Hsien T. Y. & Rorrer G. L. Effects of Acylation and Crosslinking on the Material Properties and Cadmium Ion Adsorption Capacity of Porous Chitosan Beads. Separ. Sci. Technol. 30, 2455–2475 (1995).

[b42] Xue C. & Wilson L. D. Kinetic study on urea uptake with chitosan based sorbent materials. Carbohydr. Polym. 135, 180–186 (2016). 26453866 10.1016/j.carbpol.2015.08.090

[b43] Shinde R. N. . Chitosan-transition metal ions complexes for selective arsenic(V) preconcentration. Water Res. 47, 3497–3506 (2013). 23622983 10.1016/j.watres.2013.03.059

[b44] Finnemore A. . Biomimetic layer-by-layer assembly of artificial nacre. Nat. Commun. 3, 966 (2012). 22828626 10.1038/ncomms1970

[b45] Wang Z. . Long-term fluorescent cellular tracing by the aggregates of AIE bioconjugates. J. Am. Chem. Soc. 135, 8238–8245 (2013). 23668387 10.1021/ja312581r

