# Supplementary material for: Chitosan Hydrogel Structure Modulated by Metal Ions
Source: Sci Rep. 2016 Oct 25;6:36005. doi: 10.1038/srep36005 (PMC5078770; doi:10.1038/srep36005)
Supplement: Supplementary Information [file srep36005-s1.pdf]

Supplementary Material

Chitosan Hydrogel Structure Modulated by Metal Ions

Jingyi Nie,[1, 2] Zhengke Wang,[1, 2] * Qiaoling Hu[1, 2]

[1] MoE Key Laboratory of Macromolecular Synthesis and Functionalization, Department of Polymer Science and Engineering, Zhejiang University, Hangzhou, China. [2] Key Laboratory of Adsorption and Separation Materials & Technologies of Zhejiang Province, Hangzhou, China.

Correspondence and requests for materials should be addressed to Z.K.W. (email: wangzk@zju.edu.cn).

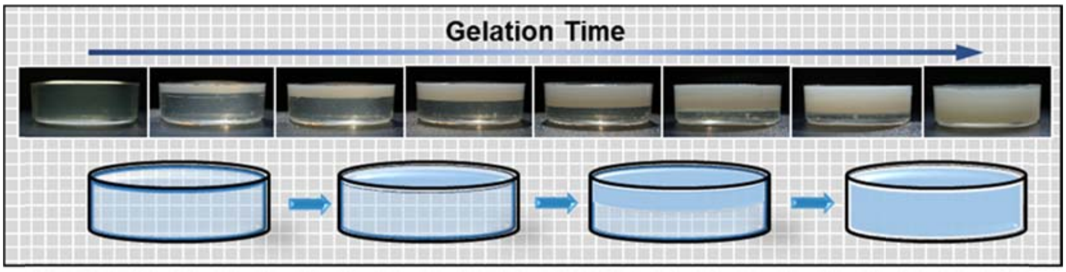

Supplementary Figure 1 | Digital photographs and scheme of the gelation process of CS hydrogel.

Gelation process of CS solution was recorded by digital photographs. Instead of forming cross-links in the whole system simultaneously, there existed a distinct gel-sol interface. OH^- diffused into the system to generate gelation gradually, and the thickness of the hydrogel increased with gelation time. So the formation of CS gel possessed a layer-wise characteristic, and brings spatiotemporal sequence to the system.

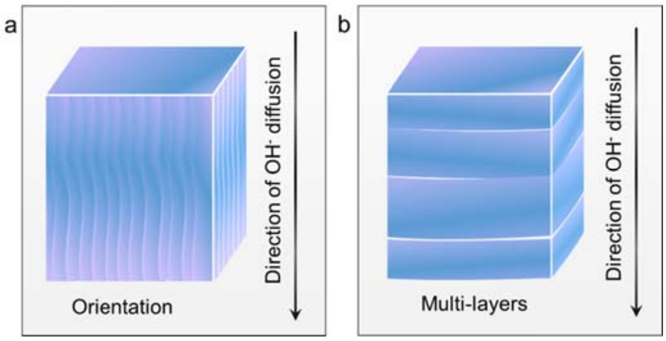

Supplementary Figure 2 | Schematic illustration of typical structure in CS hydrogel. (a) oriented structure; (b) multi-layered structure.

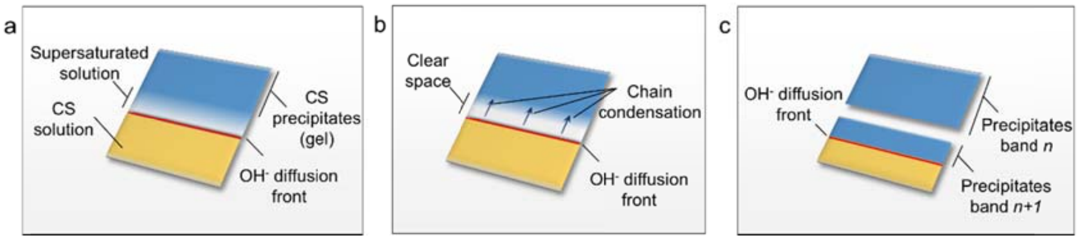

Supplementary Figure 3 | Schematic illustration of the layer formation in CS system by Liesegang Ring supersaturation theory.

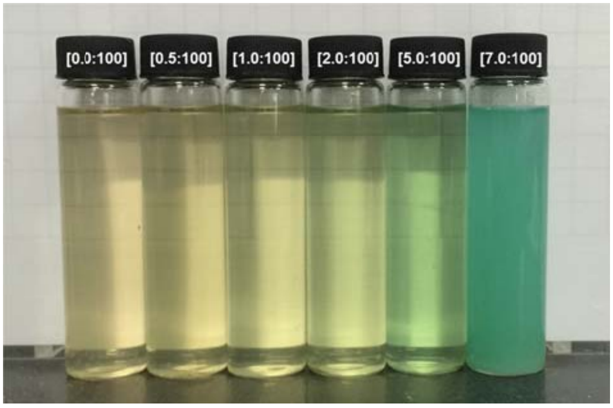

Supplementary Figure 4 | Images of Cu^{2+}-CS solution with different molar ratios of Cu^{2+} and amino group.

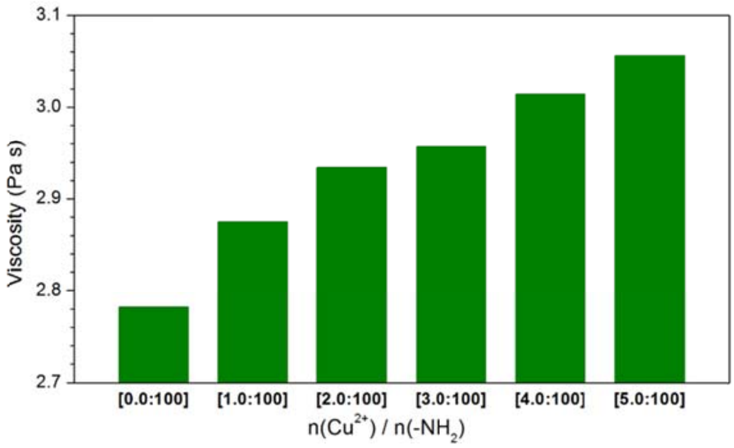

Supplementary Figure 5 | Influence of $n(Cu^{2+})/n(-NH_2)$ on the viscosity of Cu^{2+}-CS

solution.

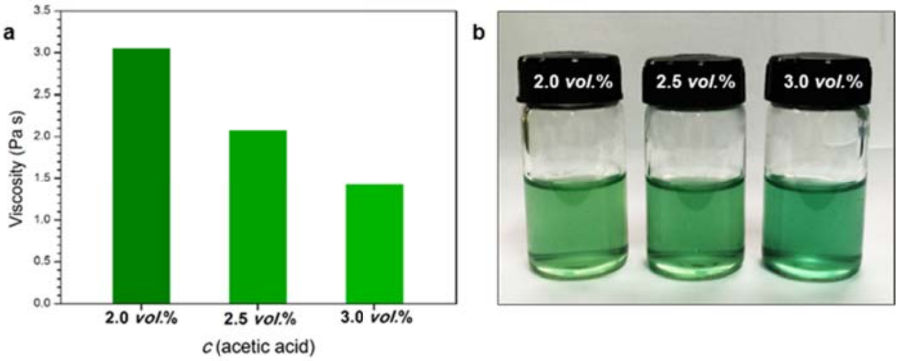

Supplementary Figure 6 | Influence of *c*(acetic acid) on the viscosity of solution.

With the increase of $c(H^+)$, the ratio of protonation of CS macromolecule increased. Thus the amount of amino groups that can form complexes with Cu^{2+} decreased, leading to the decrease of solution viscosity.

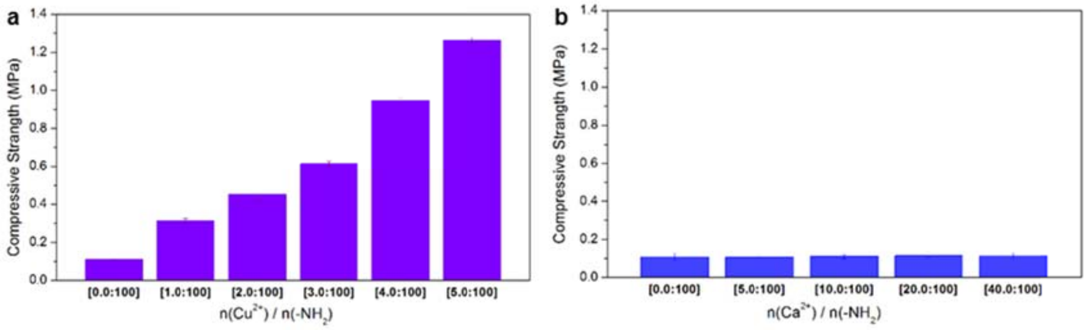

Supplementary Figure 7 | Influence of metallic ions on the strength of CS hydrogel. (a) Influence with different molar ratios of Cu^{2+} and amino group; (b) Influence with different molar ratio of Ca^{2+} and amino group.

The mechanical property of CS hydrogels were obtained on a universal materials testing machine (Instron, 5543A) at a strain rate of 2% min^{-1} for compression tests at room temperature. Hydrogels were prepared to be cylinder samples.

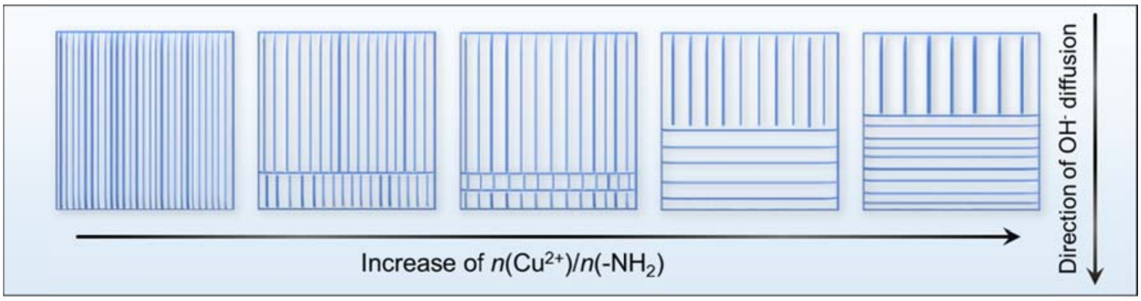

Supplementary Figure 8 | Schematic structural transition of copper-CS hydrogel with the increase of $n(Cu^{2+})/n(-NH_2)$.

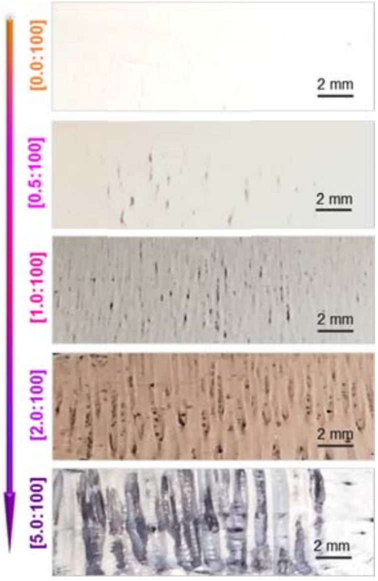

Supplementary Figure 9 | Digital images of the oriented regions in freeze-dried copper-CS hydrogels, with different molar ratios of Cu^{2+} and amino group.

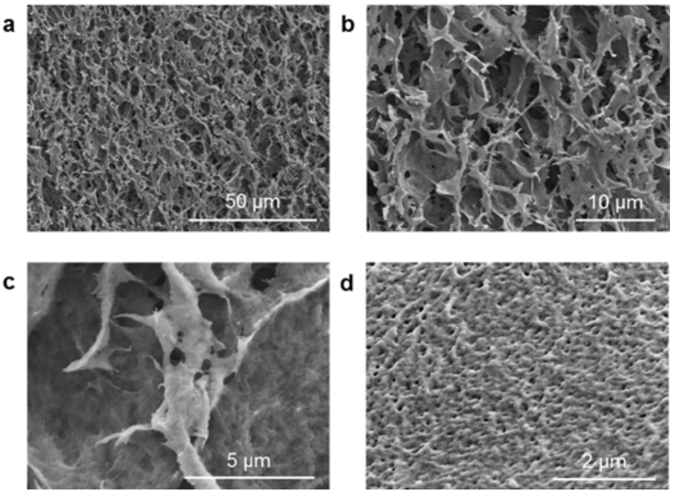

Supplementary Figure 10 | SEM images of freeze-dried copper-CS hydrogel samples, with different magnification.

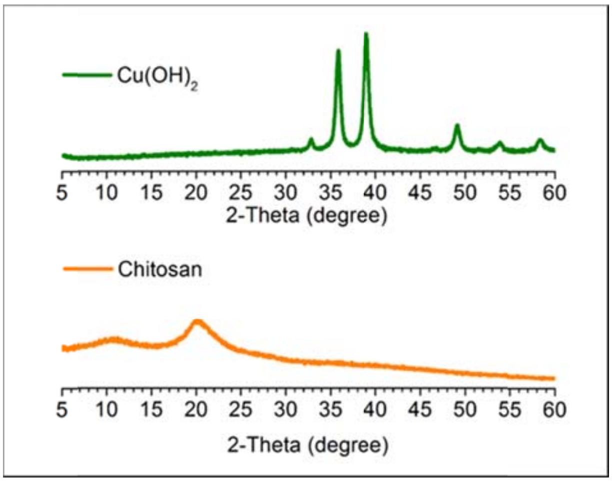

Supplementary Figure 11 | XRD profiles of $Cu(OH)_2$ and CS raw material.

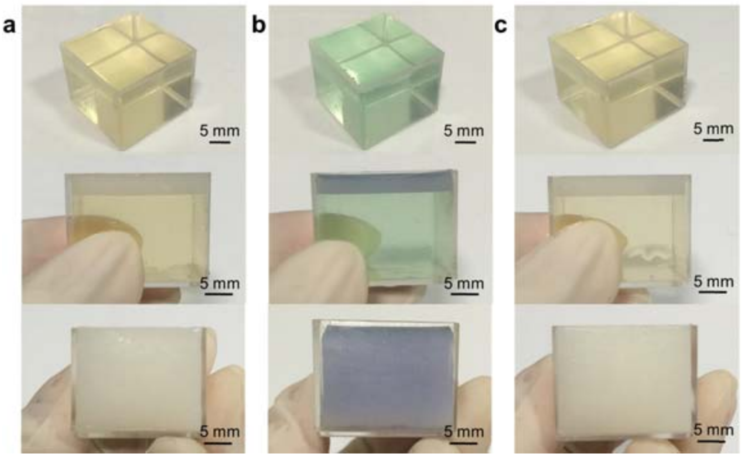

Supplementary Figure 12 | Digital images of gelation process of CS solution and CS solution with metal ions. (a) CS solution; (b) CS solution with Cu^{2+} ions, $n(Cu^{2+})/n(-NH_2)$=5.0:100; (c) CS solution with Ca^{2+} ions, $n(Ca^{2+})/n(-NH_2)$=40.0:100.

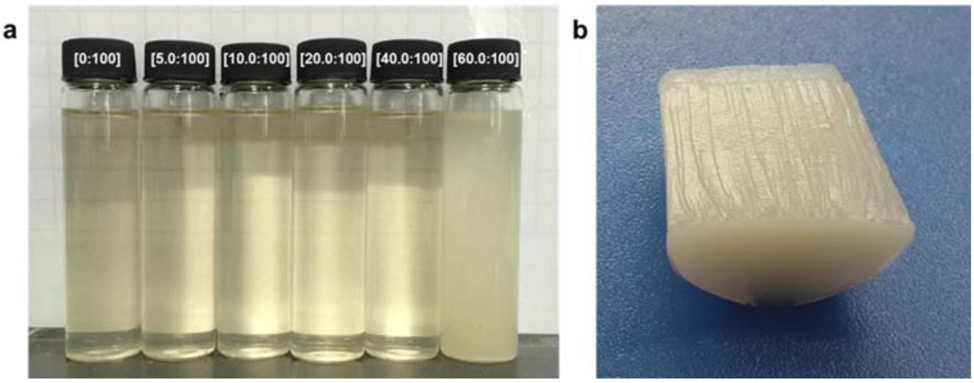

Supplementary Figure 13 | Digital image of Ca^{2+}-CS solution and calcium-CS hydrogel. (a) Ca^{2+}-CS solution with different molar ratios of Ca^{2+} and amino group. (b) The longitudinal section of calcium-CS hydrogel.

When other parameters were fixed, the solution samples were clear with $n(Ca^{2+})/n(-NH_2)$ lower than 40.0:100 (molar ratios of Ca^{2+} and amino groups), and became cloudy when $c(Ca^{2+})$ kept increasing.

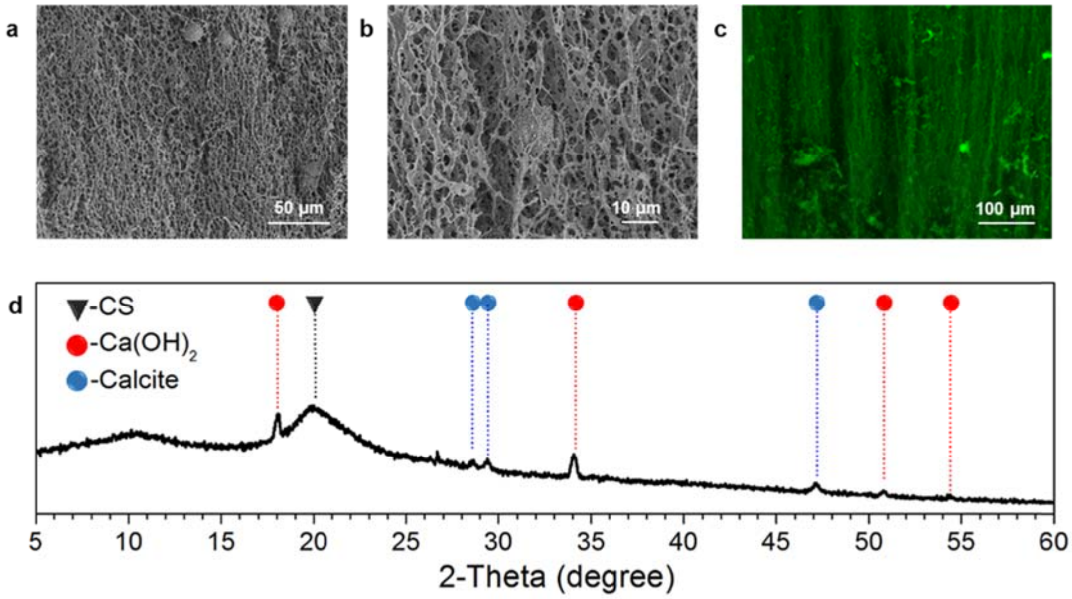

Supplementary Figure 14 | Microscopic structure of calcium-CS hydrogel, $n(Ca^{2+})/n(-NH_2)$=5.0:100. (a), (b) SEM images of freeze-dried calcium-CS hydrogel sample; (c) CLSM image of calcium-CS hydrogel; (d) XRD profile of calcium-CS hydrogel sample prepared by *in-situ* precipitation.

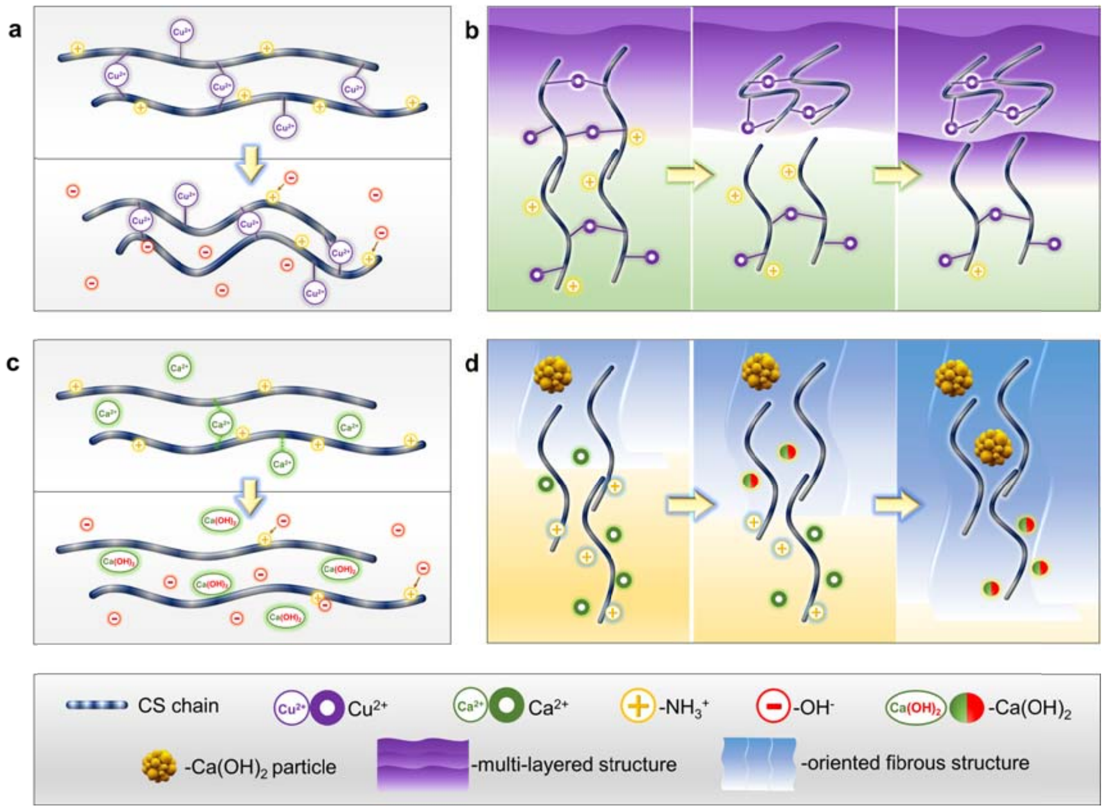

Supplementary Figure 15 | Schematic illustration of the interaction among CS and metal ions during gelation process. (a), (b) copper-CS system; (c), (d) calcium-CS system; inter/intramolecular interaction among CS chains was not depicted in this scheme for simplicity.

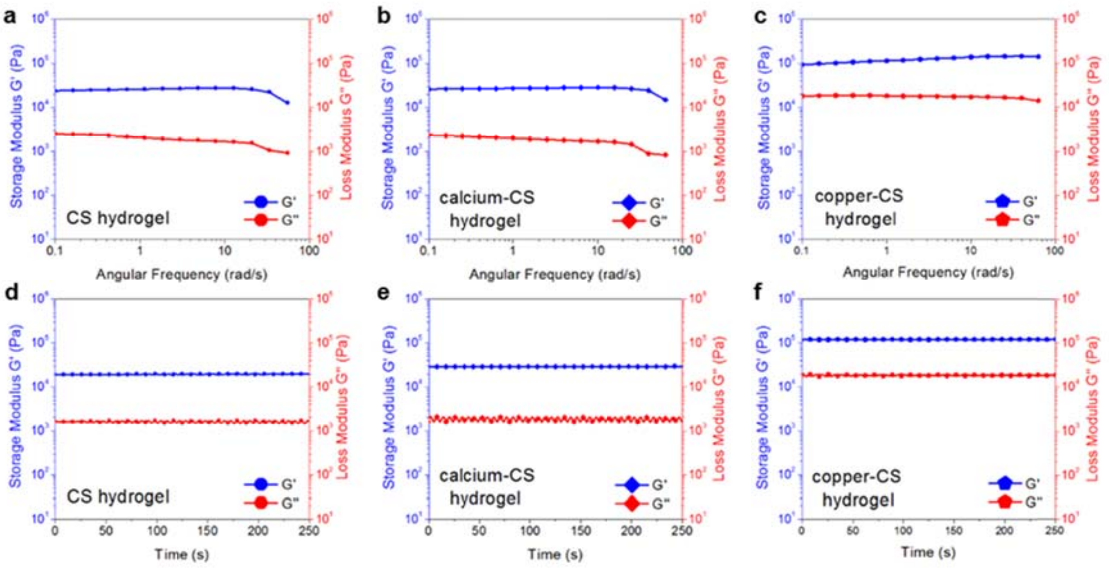

Supplementary Figure 16 | (a-c) dynamic frequency sweep of storage moduli and the loss moduli of the hydrogel: (a) pure CS hydrogel, (b) calcium-CS hydrogel, (c) copper-CS hydrogel; (d-f) dynamic time sweep of storage moduli and the loss moduli of the hydrogel: (d) pure CS hydrogel, (e) calcium-CS hydrogel, (f) copper-CS hydrogel; $n(Cu^{2+})/n(-NH_2)$=5.0:100 and $n(Ca^{2+})/n(-NH_2)$=5.0:100.

Supplementary Fig. S16 showed the rheological data of pure CS hydrogel, calcium-CS hydrogel, and copper-CS hydrogel. Dynamic frequency sweep and dynamic time sweep measurements were performed on the samples. (1) Dynamic frequency sweep of the hydrogels was performed at 0.5% strain. For all the hydrogel samples, the values of the storage modulus (G') was much higher than that of the loss modulus (G") in the scanning range. The G' was always dominant in contrast with the G", indicating

the elastic nature of these gel. (2) Dynamic time sweep of G' and G" of the hydrogel was performed at the strain of 0.5% and at the frequency of 1 rad/s. Dynamic time sweep data showed that the value of G' and G" remained constant during the entire measurement. (3) The value of G' and G" of calcium-CS hydrogel were similar to those of pure CS hydrogel, respectively. While for copper-CS hydrogel, the G' and G" value were much higher. This supported the point discussed in the main text. The introduction of metal ions that has strong affinity with CS, such as Cu^{2+} ions, leads to strong ionic cross-linking and enhanced mechanical property. On the other hand, Ca^{2+} ions have weak affinity with CS and will not increase the mechanical property apparently.
